# Cross-Sectional Analysis of Videonystagmography (VNG) Findings in Balance Disorders

**DOI:** 10.7759/cureus.34795

**Published:** 2023-02-09

**Authors:** Areej Moideen, Abhilash Konkimalla, Amit Kumar Tyagi, Saurabh Varshney, Amit Kumar, Bhinyaram Jat, Ramesh Prasath, Mangal Chandra Yadav

**Affiliations:** 1 Otolaryngology, All India Institute of Medical Sciences, Rishikesh, IND; 2 Otolaryngology - Head and Neck Surgery, All India Institute of Medical Sciences, New Delhi, IND; 3 Otolaryngology, All India Institute of Medical Sciences, Deoghar, IND; 4 Audiology, All India Institute of Medical Sciences, Rishikesh, IND

**Keywords:** nystagmus, smooth pursuit, videonystagmography, saccade, vertigo

## Abstract

Objective: To understand the videonystagmography (VNG) findings in various balance disorders in 67 patients who presented to the outpatient department of an otorhinolaryngology clinic.

Materials and methods: This cross-sectional study was conducted in the outpatient department of the otorhinolaryngology clinic of a tertiary care center. A total of 67 patients between the age group of 18 and 70 years with balance disorders were included in the study. VNG findings in different balance disorders were observed and analyzed.

Results: A total of 67 patients were enrolled in the study. Findings like caloric inversion and optokinetic nystagmus do not always indicate a central balance disorder due to technical errors and other limitations during the test. However, abnormal saccades seem to be a more relevant finding in central disorders. Rare variants of benign paroxysmal positional vertigo (BPPV) like multiple canal BPPV were also diagnosed using VNG.

Conclusion: VNG has come out as a very useful test in our study aiding in 75% of diagnoses. The overall benefits of VNG in balance disorders are immense and necessitate their inclusion in every vertigo clinic.

## Introduction

Balance disorders account for one-fourth of the referrals to ENT and neurology clinics. Detailed history and clinical examination are the gold standards for the assessment of vertigo patients. But often, clinical evaluation alone remains inconclusive. This necessitates the need for an additional effective mode of evaluation [1].

Videonystagmography (VNG) is a computerized, non-invasive, objective test that measures eye movements using infrared goggles. VNG has a better resolution of about 0.1 degrees and it can measure nystagmus as low as 0.5 degrees. The torsional component of nystagmus can also be recorded [2,3].

Studies using various findings on electronystagmography (ENG) and its pattern in different etiologies of balance disorders have been done, but studies on VNG findings in Asian literature are scarce. So far, to the best of our knowledge, there is no Indian literature on VNG findings in various balance disorders. Hence, this study was conducted to understand the role of VNG in reaching a diagnosis along with clinical examination.

## Materials and methods

The present study included 67 patients with complaints of vertigo between the age group of 18 and 70 years. Patients with any significant defective vision, acute illness, or diagnosed neurological disorders or claustrophobia were excluded from the study.

Interacoustics VN415/VO425 (Interacoustics A/S, Middelfart, Denmark) VNG machine was used in our study. Before conducting a VNG, standard precautions were followed.

VNG consists of multiple tests that intend to test the functionality of the vestibular system. It is able to differentiate between peripheral and central disorders and the side of the lesion. It includes tests to check the oculomotor system and the vestibulo-ocular reflex. It helps to record, analyze, and report eye movements using video imaging technology.

Before conducting a VNG, it is essential to take a detailed history and clinical examination, including otoscopy and ocular movements, followed by a neuro-otological examination. It is important to ask the patient not to have any eye makeup or contact lenses during the test. Patients are also advised to have food at least three hours prior to the test, or else they are prone to develop nausea and vomiting during the tests. Patients should be briefed about the nature of the test being benign, or else erroneous results may occur if the patient is apprehensive/anxious. Drugs like benzodiazepines, anti-depressants, vestibular sedatives, and anxiolytics suppress the caloric test. Neck disorders like ankylosing spondylitis should be ruled out before the test because in such cases, static and dynamic positional tests are contraindicated. Ear examination should be done in all patients to assess the status of the tympanic membrane and to remove any wax or debris present. Any ptosis, refractory error, or restricted eye movements should be documented, and, in such cases, binocular VNG should be avoided. One must ensure that the patient is off vestibular sedatives for a minimum of 48-72 hours, and once the prerequisites are met, one can proceed with the calibration of the eye for the test. The parameters assessed during the test are listed in Table 1.

**Table 1:** Parameters assessed in VNG VNG: videonystagmography; SPV: slow phase velocity; TN: temporo-nasal; NT: naso-temporal.

S. No.	Test	Parameters
1	Spontaneous nystagmus	SPV
2	Gaze	SPV
3	Smooth pursuit	Gain (%)
4	Saccade	Latency velocity precision (%)
5	Optokinetic nystagmus	Direction gain (%), asymmetry index (TN/TN + NT)
6	Static and dynamic, positional nystagmus	SPV
7	Caloric test	Caloric weakness (%), directional preponderance (%), fixation index

Statistical analysis

Categorical variables were presented in numbers and percentages. Continuous variables were presented as mean, SD, and median. Qualitative variables were compared using the chi-square test and Fisher's exact test. A p-value of <0.05 was considered statistically significant. The data were entered in a Microsoft Excel spreadsheet (Microsoft Corporation, Redmond, WA) and analysis was done using IBM Statistical Package for Social Sciences (SPSS) software version 21 (IBM Corp., Armonk, NY). Analysis was done using descriptive and inferential statistics.

## Results

The age of the patients who were included in the study ranged from 18 to 63 years. The mean age was 42.2 ± 10.6 years, with a male-to-female ratio of 1.16:1.00. The comorbidities seen in our study were dyslipidemia (16.4%), hypertension (8.9%), hypothyroidism (7.4%), diabetes mellitus (5.9%), and isolated cases of seizure disorder (1.4%) and vitamin B12 deficiency (1.4%). The balance disorders presented to our department were classified based on etiology into peripheral, central, functional, and metabolic causes (Table 2).

**Table 2:** Etiology of balance disorders

Etiology		Number (%)
Peripheral		37 (55.2)
Central		23 (34.3)
Functional	Chronic subjective dizziness	02 (02.9)
	Psychogenic vertigo	02 (02.9)
Metabolic	Hypothyroidism	02 (02.9)
	Dyslipidemia	01 (01.4)
Total		67 (100)

Tinnitus was the most commonly associated symptom in patients with balance disorders and was a statistically significant finding in patients with peripheral disorders (Fisher's exact test, p < 0.002).

**Figure 1:**
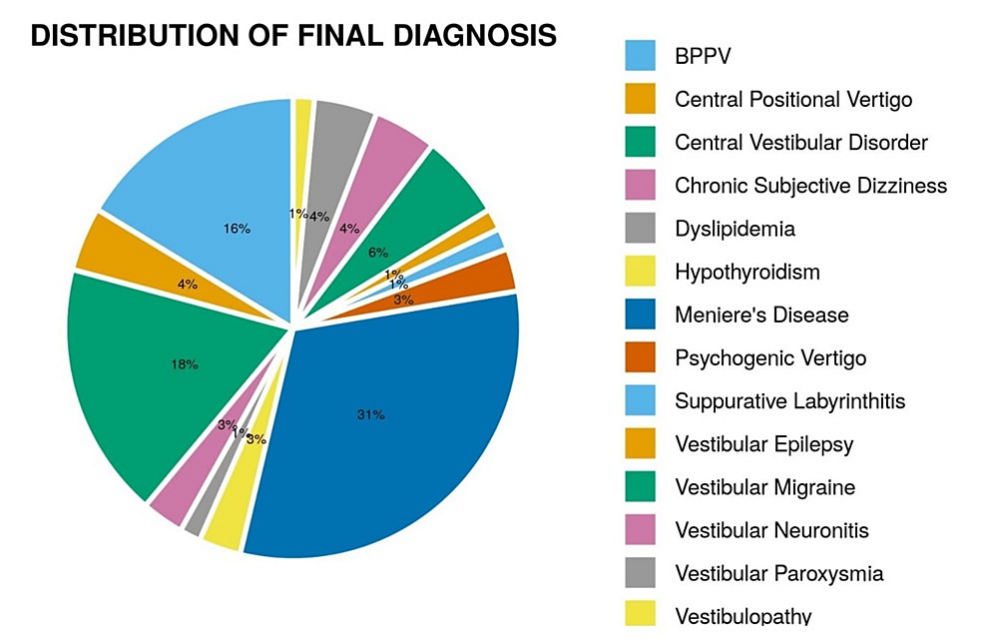
Distribution of final diagnosis BPPV: benign paroxysmal positional vertigo.

The final summary of various diagnoses of balance disorders and their VNG findings are given in Figure 1 and Table 3.

**Table 3:** Summary of VNG findings in balance disorders VNG: videonystagmography; BPPV: benign paroxysmal positional vertigo; VM: vestibular migraine; CPN: central positional nystagmus; CSD: chronic subjective dizziness.

	Disease	Spontaneous nystagmus	Abnormal gaze	Abnormal smooth pursuit	Abnormal saccade	Static positional nystagmus	Abnormal optokinetic nystagmus	Positive Dix-Hallpike test	Abnormal caloric test
1	Meniere's disease (n = 21)	01 (4.7%)	01 (4.7%)	02 (9.5%)	04 (19%)	05 (23.8%)	12 (57.1%) (P = 0.031)	03 (14.2%)	7/15 (46.6%)
2	Central vestibular disorder (n = 12)	-	-	07(58.3%) (P = 0.002)	09(75%) (P < 0.001)	02 (16.6%)	08 (66.6%)	02 (16.6%)	1/10 (10%)
3	BPPV (n = 11)	-	-	01 (9%)	-	05 (45.4%) (P = 0.013)	06 (54.5%)	04 (36.3%)	2/8 (25%)
4	VM (n = 4)	-	-	-	01 (25%)	01 (25%)	03 (75%)	-	2/3 (6.6%)
5	Vestibular paroxysmia (n = 3)	-	-	-	01 (33.3%)	01 (33.3%)	-	01 (33.3%)	1/1 (100%)
6	CPN (n = 3)	-	-	02 6.6%)	03 (100%) (P = 0.017)	03 (100%) (P = 0.018)	03 (100%)	02 (66.6%) (P = 0.047)	1/3 (33.3%)
7	Vestibular neuronitis (n = 3)	-	-	-	-	02 (66.6%) (P = 0.031)	03 (100%)	01 (33.3%)	3/3 (100%) (P = 0.041)
8	CSD (n = 2)	-	-	01 (50%)	-	-	01 (50%)	-	-
9	Psychogenic dizziness (n = 2)	-	-	-	-	-	01 (50%)	-	0/1 (0%)
10	Vestibular epilepsy (n = 1)	-	-	01 100%)	01 (100%)	-	01 (100%)	-	1 (100%)
11	Unilateral Vestibulopathy (n = 1)	-	-	-	-	-	-	-	1 (100%)
12	Suppurative labyrinthitis (n = 1)	-	-	-	-	01 (100%)	-	01 (100%)	1 (100%)
13	Hypothyroidism (n = 2)	-	-	-	-	-	01 (100%)	-	0/1 (0%)
14	Dyslipidemia (n = 1)	-	-	-	-	-	01 (100%)	-	0/1 (0%)

VNG in balance disorders

The various tests applied to the study population yielded the following results in 67 patients. Optokinetic nystagmus (OKN) was the most common abnormality noted. Caloric tests could not be done in 18 patients due to technical errors and lack of cooperation. The most common trinary code noted was 0000. Caloric inversion was seen in five patients.

Meniere’s Disease

There were 21 patients with Meniere’s disease. The most common abnormality noted was in OKN, which was significant (Fisher's exact test, p = 0.031). Hypoactive labyrinth was noted in seven out of 15 patients (46.6%). Five patients (23.8%) had positive static positional nystagmus in different positions and three patients (14.2%) had positive Dix-Hallpike test. Using VNG, we could diagnose three patients with secondary benign paroxysmal positional vertigo (BPPV).

BPPV

There were 11 patients with BPPV, of which there were five cases of the posterior semicircular canal (PSCC) BPPV, four cases of the lateral semicircular canal (SCC) BPPV, and a single case of the anterior semicircular canal (ASCC) BPPV and multiple canal BPPV (anterior and lateral).

Vestibular Neuronitis

There were three patients with vestibular neuronitis, and caloric weakness was significantly abnormal in all cases (Fisher's exact test, p = 0.041).

Unilateral Vestibulopathy

A single case of unilateral vestibulopathy was present who had a 37% unilateral weakness in the caloric test.

Suppurative Labyrinthitis

Only patients with post-traumatic suppurative labyrinthitis had a secondary PSCC BPPV observed during the test.

Central Vestibular Disorders

There were 12 patients with central vestibular disorders in our study. There were statistically significant anomalies in smooth pursuit noted in seven patients (58.3%) (Fisher’s exact test, p = 0.002) and abnormal saccade in nine patients (75%) (Fisher’s exact test, p < 0.001). Asymmetry in OKN was present in eight (66.7%) patients. An abnormal fixation index in the caloric test was present in three (33.3%) out of nine patients.

Vestibular Migraine

Among four cases, abnormalities in saccade, static positional nystagmus, and OKN were observed, and a hypoactive labyrinth was noted in two cases.

Vestibular Paroxysmia

There were three patients with vestibular paroxysmia, of which only one patient could undergo a caloric test and had a hypo-functioning labyrinth. A unilateral increase in the latency of saccade was observed in a single patient. Positional nystagmus was present in two patients (66.6%), of which one patient had persistent positional nystagmus (33%).

Central Positional Nystagmus

There were three cases of CPN, of which all three patients (100%) had statistically significant abnormal saccades (Fisher’s exact test, p = 0.017). Static positional nystagmus and positive Dix-Hallpike test present in two patients (66.6%) were also significant (Fisher’s exact test, p = 0.018 and 0.047, respectively).

## Discussion

The most common age group affected in our study was between 40 and 49 years (24/67), with 35.8% of total patients. Our results are in accordance with the studies done by Abrol et al. (2001), concluding 40-59 years (47.8%) as the most common age group, Burman et al. (2002), concluding 31-50 years (65.2%) as the most common age group, Gupta (2015), concluding 41-60 years (42.3%) as the most common age group, and Sonawale et al. (2018), concluding 41-60 years (38%) as the most common age group [4-7].

Based on etiology, balance disorders were classified into central, peripheral, functional, and metabolic categories. The most common etiology observed in our study was peripheral seen in 37 patients (55.2%), followed by central in 23 patients (34.3%). Vertigo clinics run by otorhinolaryngology specialists note a high number of patients with peripheral disorders. In our study, Meniere’s disease was the most common peripheral disorder diagnosed in 21 patients (31.3%), followed by BPPV in 11 patients (16.4%). This is in discordance with the majority of previous studies that stated BPPV as the most common peripheral disorder [5,8]. This might be due to the fact that most patients of BPPV were diagnosed clinically at the time of presentation in the outpatient department and underwent corrective positional maneuvers simultaneously and hence not included in the study for VNG examination. However, Meniere’s disease was also one of the common etiologies reported by Zorengpuii et al. at 28% and Cawthorne et al. at 2.54-63.8% [9].

We observed that tinnitus was the most consistent symptom seen in 58.2% of patients. Tinnitus was also strongly associated with peripheral vertigo when compared to other groups and this correlation was statistically significant (p = 0.002). Gopinath et al. (2009) [10] concluded that tinnitus had a stronger association with peripheral vertigo rather than hearing loss. Our study suggests that tinnitus is a more sensitive marker than hearing loss when one assesses cochlear involvement in patients with vertigo.

Of the patients with peripheral vertigo, 23 (62.2%) had unilateral sensory neural hearing loss (SNHL) in our study. A higher incidence of unilateral SNHL in peripheral vertigo can be attributed to the greater number of patients with Meniere’s disease in our study who developed cochlear dysfunction due to progressive disease activity (endolymphatic hydrops).

**Figure 2:**
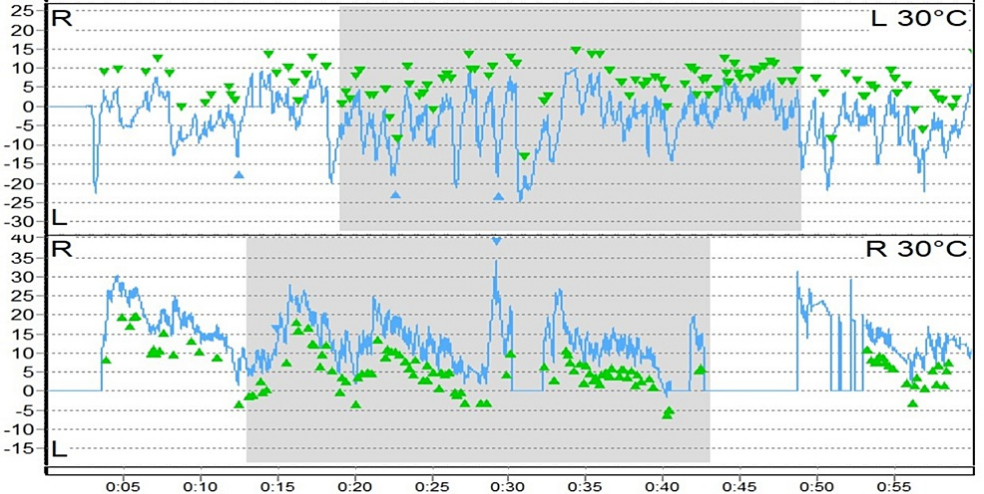
Caloric inversion (depicted by green arrowheads)

Caloric inversion, a rare finding in VNG and indicative of brainstem pathology, was noted in five patients (7.4%). Caloric inversion can also be due to technical errors like using air caloric tests in subjects with tympanic membrane perforation. We presume the abnormal findings were due to technical errors, the nature of which could not be explained (Figure 2) [11,12].

OKN was the most common abnormal test noted. Forty patients (59.7%) had asymmetry in OKN. Abnormal OKN is an indicator of central vertigo; however, we noticed abnormal OKN in only 14/23 patients of central etiology and we noted this in other etiologies too (26/44) [12]. This discrepancy may be due to the strenuous nature of the test. Further lack of understanding about performing the test by the patient can also yield erroneous results. Hence, abnormal OKN solely does not necessarily lead to a diagnosis of central vestibular disorder, as suggested by various studies. However, abnormal saccades were the most common finding in central etiology (15/23). Mccaslin et al. (2009) also concluded that saccade and gaze stability tests are the most diagnostically useful and pursuit test and OKN are of less diagnostic use. They also concluded that instead of true reflexive, brain-stem-mediated "stare" OKN, most ENG/VNG systems depict only volitional "look" OKN representing the pursuit eye movement system [13].

VNG findings in various balance disorders

Nearly half of the patients with Meniere’s disease (seven out of 15) had a hypo-functional labyrinth with varying degrees of compensation. These results are consistent with previous literature by Carlos et al. (2002), where 50% of study patients had unilateral hypo-functioning labyrinth and the rest had normal caloric tests, Güneri et al. (2016), where the hypo-functioning labyrinth was noted in 50-67% and normal labyrinth in 6-11%, and Oliveira et al. (2021), where the hypoactive labyrinth was noted in 56.4% of symptomatic Meniere’s disease patients and 36% of asymptomatic Meniere’s disease patients [14-16].

The occurrence of secondary BPPV in Meniere’s disease was studied by Eckhard et al. (2019), who concluded that unilateral PSCCs are more commonly affected [17]. In comparison to idiopathic BPPV, patients with BPPV secondary to Meniere’s disease were prone to have higher recurrence rates and longer duration of symptoms.

Abnormal gaze nystagmus, smooth pursuit, and saccade were present in a patient who was a case of congenital Brown’s syndrome with Meniere’s disease described by Wright (1999) [18,19]. We observed that eliciting OKN at a higher velocity (50˚/s) resulted in abnormal results in 11 (64.7%) patients. Patients had difficulty performing the test at higher velocities leading to abnormal values. Oculomotor tests are not known to produce abnormal findings in peripheral vertigo. Abnormal OKN in our study could be either due to the age of these patients (all aged more than 40 years) or lack of attention or decreased mentation in the post-prandial state or inadequate visual field coverage of the OKN stimuli [13,20,21].

**Figure 3:**
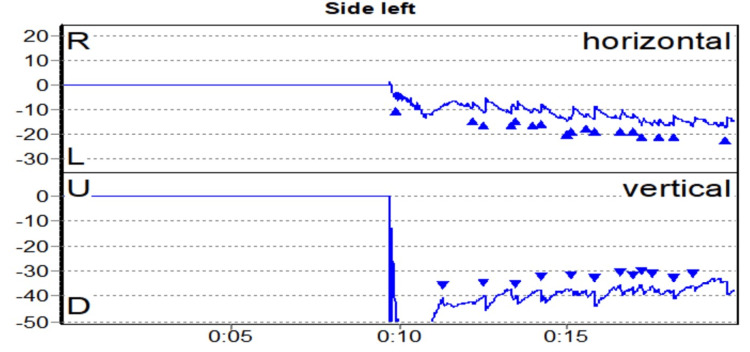
Multiple canal BPPV BPPV: benign paroxysmal positional vertigo.

**Figure 4:**
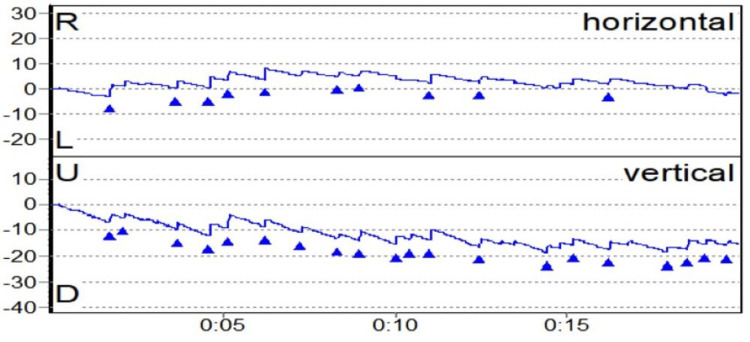
Right upbeating and torsional (anti-clockwise) nystagmus in a case of right PSCC BPPV PSCC: posterior semicircular canal; BPPV: benign paroxysmal positional vertigo.

**Figure 5:**
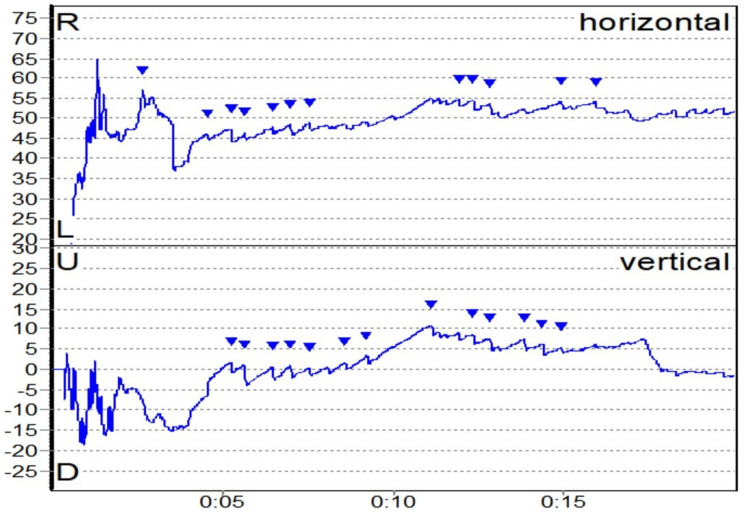
Left downbeating and torsional (clockwise) nystagmus in a case of left ASCC BPPV ASCC: anterior semicircular canal; BPPV: benign paroxysmal positional vertigo.

BPPV was the second most common peripheral vestibular disorder present in 11 patients (18.05%). The trend we noticed is similar to previous studies that suggest that BPPV in posterior SCC is the commonest, followed by lateral SCC and rarely anterior SCC, as suggested by Maslovara et al. (2014), where 97.3% had posterior SCC and 2.7% had lateral SCC [22]. Multiple canal BPPV usually affects bilateral PSCC as was observed by Balatsouras (2012) [23]. However, one patient in our study had simultaneous involvement of anterior and lateral SCCs, which is extremely rare (Figures 3-5).

Vestibular neuronitis was the next common entity in peripheral vestibular disorders seen in three (4.2%) patients. Due to the smaller number of subjects in this category, generalizing the findings (of caloric weakness with normal fixation index) is not prudent. None of the patients had significant hearing loss, hence pointing to a diagnosis of vestibular neuronitis [24].

**Figure 6:**
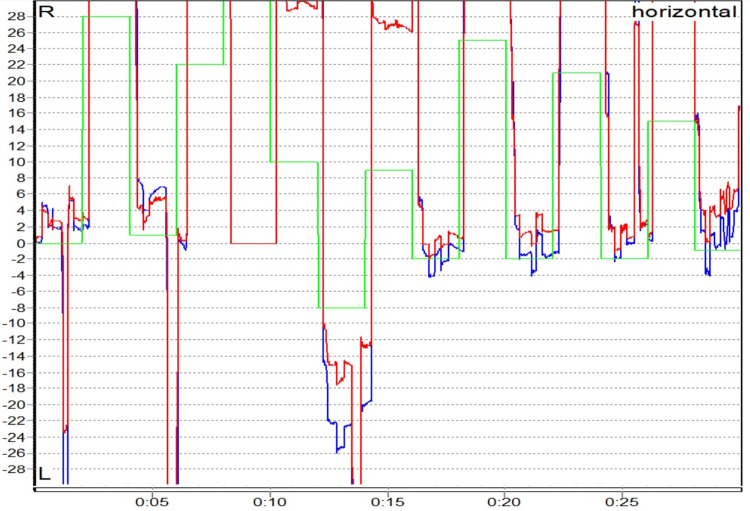
Abnormal saccade (hypermetria) in a case of central vestibular disorder

**Figure 7:**
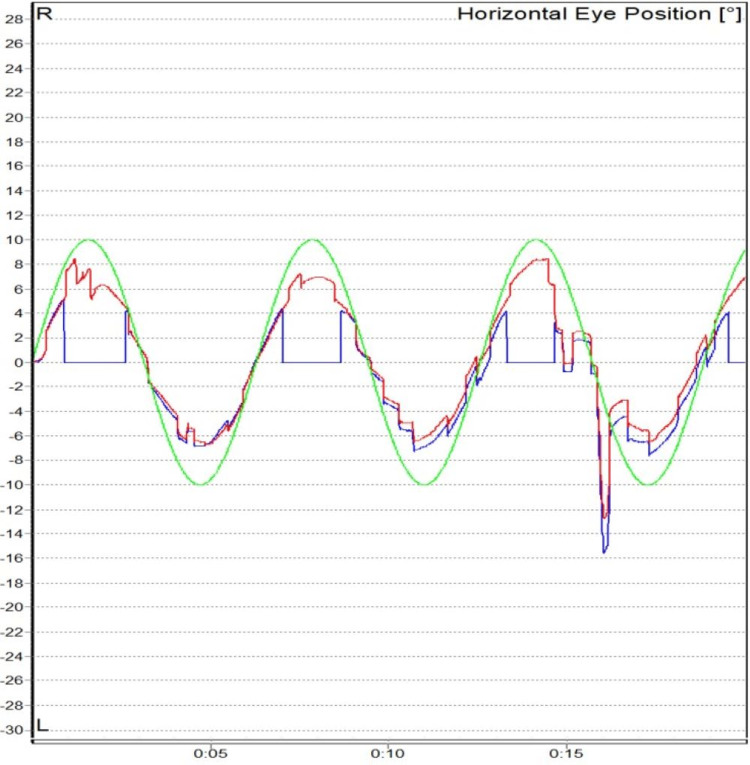
Abnormal smooth pursuit (gain) in a case of central vestibular disorder

Central vestibular disorders were the most common cause of central vertigo in our study with 12 (17.9%) patients. Abnormal saccades, smooth pursuit, and OKN were noted in our study. It is well known from previous studies that abnormalities in oculomotor tests indicate a central pathology, and it needs further investigation to localize the site of the defect as suggested by Karatas (2008) and Mohammed (2016) (Figures 6, 7) [25,26].

In view of the wide range of clinical presentations of central vestibular disorders, VNG alone cannot lead to a specific diagnosis. Hence, all suspected cases of central vertigo should undergo additional investigations like vertebral artery screening test (VAST), carotid, vertebrobasilar Doppler, and brain MRI. Mostafa et al. (2014) proposed a diagnostic accuracy of 40% when combining additional tests with VNG [27]. All our patients with central vestibular disorders were diagnosed on the basis of history, clinical examination, and VNG.

Vestibular migraine was present in four (5.6%) patients. Patients were diagnosed based on Neuhauser and Lempert’s criteria. Our results are in accordance with multiple previous studies on vestibular migraine. According to Celebisoy et al. (2008), patients with vestibular migraine had abnormal oculomotor and caloric tests. Polensik and Tusa (2010) reported spontaneous nystagmus in 19% with a normal bithermal caloric test. Audiological and vestibular assessment done by Lepcha et al. (2015) observed that 16% of patients had SNHL and 64% of patients had caloric function abnormalities [28-30]. Sensory neural hearing loss was observed in two (50%) of our patients. Mathew et al. (2016) observed hearing loss in 33% of patients with vestibular migraine. Hearing loss in patients with vestibular migraine is due to the vasospasm of labyrinthine arteries as suggested by Kırkım et al. (2017) [31].

Vestibular paroxysmia was observed in three patients (4.2%) among the study population. Diagnosis of vestibular paroxysmia was based on the criteria laid down by the Classification Committee of the Bárány Society [32]. According to Choi et al. (2018), there is cross-compression of the vestibular nerve leading to a hypoactive labyrinth of the affected side and hearing loss [33]. All three patients had moderate to severe SNHL on the affected side of the vascular loop noted in the MRI (right type 1 anterior inferior cerebellar artery (AICA), left type 1 AICA, and bilateral AICA loop). Our results are consistent with Ihtijarevic et al. (2019), who reported nystagmus directed toward the side of the loop, with a hypoactive labyrinth on the side of the loop, and the vessel involved was AICA [34].

Central positional nystagmus was diagnosed in three patients (4.2%) based on the criteria proposed by Büttner et al. (1999) [35]. The results in our study are consistent with those in recent literature by Macdonald et al. (2017) [36].

There was an isolated case of vestibular epilepsy in our study. Abnormal smooth pursuit and saccade (hypermetria) with caloric weakness were observed in this patient. According to Hamed et al. (2017), patients with epilepsy can have vestibulopathies. They observed anomalies in spontaneous nystagmus (2.2%), smooth pursuit (42.2%), saccade (44.4%), OKN (42.2%), and canal weakness (44.4%) in 45 patients. They concluded that these vestibular anomalies could be due to the permanent damaging effect of epilepsy on the vestibular cortical area or due to anti-epileptic medication like carbamazepine. However, due to a limited number of cases, a generalized pattern cannot be described [37].

Central disorders tend to have more abnormal findings in oculomotor tests like saccades, smooth pursuit, or gaze, unlike peripheral disorders, which tend to have abnormal findings in positional tests or caloric tests. To differentiate between central and peripheral nystagmus in positional tests, the period of latency to develop nystagmus and to suppress nystagmus with fixation of vision can be used.

Limitations

Bithermal caloric tests could not yield results in 18 patients due to technical error or lack of compliance; however, these patients were not excluded from the study as they yielded significant findings in other VNG tests.

## Conclusions

VNG has come out as a very useful test in our study aiding in 75% of diagnoses. However, many abnormal findings did not always fit into the clinical scenario. Findings like caloric inversion and OKN do not always indicate a central balance disorder due to technical errors and other limitations during the test. However, abnormal saccades seem to be a more relevant finding in central disorders. Rare variants of BPPV, such as multiple canal BPPV, were also diagnosed using VNG. Central disorders tend to have more abnormal findings in oculomotor tests like saccades, smooth pursuit, or gaze, unlike peripheral disorders, which tend to have abnormal findings in positional tests or caloric tests. To differentiate between central and peripheral nystagmus in positional tests, the period of latency to develop nystagmus and to suppress nystagmus with fixation of vision can be used. Further, suspected cases of central vertigo should undergo additional investigations along with detailed history, neurotological examination, and VNG. However, to overcome the low false positive of VNG findings, it is essential to interpret and rationalize the findings of VNG with adequate knowledge of the behavior of vertigo syndromes. The overall benefits of VNG in balance disorders are immense and necessitate their inclusion in every vertigo clinic.
